# Advances in the prevention and treatment of radiation-induced brain necrosis: a narrative review

**DOI:** 10.3389/fonc.2026.1780434

**Published:** 2026-06-29

**Authors:** Zhihong Zhao, Wan He, Jinquan Xia, Xiangcheng Wang, Guixiang Liao

**Affiliations:** 1 Department of Kidney Disease and Urology, Shenzhen People’s Hospital (The Second Clinical Medical College, Jinan University, The First Affiliated Hospital, Southern University of Science and Technology), Shenzhen, China; 2 Department of Oncology, Shenzhen People’s Hospital (The Second Clinical Medical College, Jinan University, The First Affiliated Hospital, Southern University of Science and Technology), Shenzhen, China; 3 Department of Nuclear Medicine, Shenzhen People’s Hospital (The Second Clinical Medical College, Jinan University, The First Affiliated Hospital, Southern University of Science and Technology), Shenzhen, China

**Keywords:** neuroprotective, pathogenesis, radiation necrosis, radiotherapy, treatment

## Abstract

Radiation-induced brain necrosis (RBN) is a serious and often debilitating complication of radiotherapy for intracranial and head and neck malignancies, with an incidence of 5–25%. It can lead to significant cognitive impairment, neurological deficits, and increased mortality. As radiotherapy techniques advance and patient survival improves, effective prevention and management of RBN have become critical clinical priorities. This review systematically summarizes the current understanding of RBN pathogenesis, highlighting the central roles of vascular injury (endothelial damage, HIF-1α/VEGF dysregulation) and neuroinflammation (microglial activation, cytokine release). We discuss recent advances in preventive strategies, including refined radiotherapy modalities such as intensity-modulated radiotherapy (IMRT), fractionated stereotactic radiosurgery (fSRS), and FLASH ultra-high-dose-rate radiotherapy, as well as pharmacological prophylaxis using bevacizumab and neuroprotective agents (NGF, GM1, edaravone). Established and emerging treatment options are reviewed, including corticosteroids, bevacizumab, hyperbaric oxygen therapy (HBOT), laser interstitial thermal therapy (LITT), and Colony Stimulating Factor 1 Receptor(CSF1R) inhibitors. We also cover innovations in imaging for early detection and differential diagnosis, including magnetic resonance spectroscopy (MRS), perfusion-weighted imaging (PWI), and Positron Emission Tomography-Computed Tomography(PET/CT) with advanced tracers (^11^C-methionine, ^18^F-FET). Finally, we summarize updates to international management guidelines, particularly the 2022 DEGRO guidelines, and propose future directions for research and therapeutic optimization.

## 1 Introduction

RBN remains a formidable challenge in neuro-oncology and radiation oncology. It occurs most commonly in patients treated for brain metastases (accounting for approximately 50–60% of RBN cases), followed by primary brain tumors such as glioblastoma and astrocytoma, and head and neck malignancies, particularly nasopharyngeal carcinoma (NPC), where the temporal lobes are in close proximity to the radiation field (1–3).The typical latency period for RBN development ranges from 3 to 12 months after conventional radiotherapy, although late-onset RBN can occur >2 years following treatment, particularly after stereotactic radiosurgery (SRS) (1, 3, 4). On conventional Magnetic Resonance Imaging(MRI), RBN classically demonstrates contrast-enhancing lesions with surrounding vasogenic edema and mass effect. Key radiographic features include a “soap bubble” or “swiss cheese” appearance (non-uniform enhancement), white matter necrosis, and the absence of discrete tumor nodules. On Computed Tomography(CT), RBN may appear as hypodense areas with variable contrast enhancement. Regarding clinical presentation, approximately 30–50% of RBN cases are asymptomatic and detected incidentally on surveillance imaging, while 50–70% are symptomatic, presenting with headaches, focal neurological deficits (e.g., hemiparesis, aphasia), cognitive impairment, or seizures (3, 5). Symptomatic RBN can significantly affect patient quality of life and survival. Key clinical challenges include: (1) diagnostic dilemma: distinguishing RBN from tumor progression remains difficult, as both can coexist and share imaging features; conventional MRI alone has limited specificity (~60%); (2) treatment resistance: some patients fail to respond to first-line corticosteroids or bevacizumab; (3) recurrence after response: 50–70% of bevacizumab-treated patients experience regrowth after discontinuation; (4) lack of standardized protocols: significant practice variation exists regarding dosing, duration, and sequencing of therapies.

Currently available treatment strategies span a spectrum from conservative management to aggressive intervention. Asymptomatic RBN typically warrants observation with serial imaging. Symptomatic RBN is managed with a stepwise approach: first-line options include corticosteroids (short-term edema control) and bevacizumab (blocking Vascular Endothelial Growth Factor(VEGF) therapy with high response rates). Second-line therapies include hyperbaric oxygen therapy (HBOT) and laser interstitial thermal therapy (LITT). Third-line/refractory options include surgical resection (necrotomy) and emerging agents such as CSF1R inhibitors.

This review aims to synthesize contemporary insights into RBN pathophysiology and the evolving landscape of strategies for its prevention and treatment.

## 2 Review methodology

This narrative review synthesizes current knowledge on the prevention and treatment of RBN based on the authors’ expertise and a focused, non-systematic literature review. The primary aim is to provide a broad, clinically oriented overview of key advances in pathogenesis, preventive radiotherapy techniques, pharmacological prophylaxis, therapeutic interventions, and imaging innovations. A comprehensive systematic literature search following PRISMA guidelines was not performed. Instead, relevant peer-reviewed articles were identified through targeted searches of the PubMed database (January 2000 – April 2026) using combinations of keywords including “radiation necrosis,” “brain necrosis,” “radiotherapy,” “proton therapy,” “radionuclide therapy,” “bevacizumab,” “LITT,” “FLASH,” and “imaging.” Priority was given to high-impact clinical trials, recent systematic reviews, and established clinical guidelines (e.g., DEGRO, NCCN). Reference lists of retrieved articles were also manually screened for additional relevant studies. Given the narrative design, this review does not include formal quality assessment or quantitative synthesis of the included evidence.

## 3 Unraveling the pathogenesis of radiation necrosis: recent insights

The precise mechanisms driving the development of RBN remain incompletely understood; however, current research has identified vascular injury and glial cell dysfunction as the primary contributing factors.

### 3.1 The role of vascular injury: updated perspectives

The vascular injury theory posits that ionizing radiation directly damages the endothelial cells lining blood vessels in the brain. This initial damage triggers a cascade of events, including fibrinoid necrosis, the formation of microthrombi, and a disruption of the critical blood-brain barrier(BBB) (6). The long-term consequences of radiotherapy often manifest as significant damage to the vascular endothelium, leading to obliterative fibrosis and increased wall thickness in irradiated blood vessels (7). Clinically, these radiation-induced vascular changes can present unique challenges during surgical procedures performed in previously irradiated fields (7).The obliterative fibrosis that develops in irradiated tissues leads to loss of normal tissue planes, making sharp dissection difficult and increasing the risk of inadvertent injury to adjacent eloquent brain structures (1, 8). Furthermore, increased vascular wall thickness combined with fragile, hyalinized vessels creates a paradoxical situation: vessels are less pliable and more difficult to dissect, yet they remain highly prone to tearing with minimal manipulation. Intraoperative hemostasis is particularly challenging because irradiated vessels exhibit impaired vasoconstriction and reduced contractile response to conventional hemostatic agents. Additionally, microthrombi within smaller vessels can obscure intraoperative visualization and complicate the distinction between viable tissue and necrotic debris. These factors collectively contribute to higher rates of postoperative complications, including intracerebral hemorrhage, delayed wound healing, and cerebral edema exacerbation, in patients undergoing necrotomy or tumor resection in previously irradiated fields (6, 8).

The overexpression of hypoxia-inducible factor-1α (HIF-1α) and VEGF plays a crucial role in exacerbating the vascular damage. In response to radiation-induced hypoxia around the tumor tissue, microglia express HIF-1α (6). Subsequently, reactive astrocytes increase the expression of VEGF, a pro-angiogenic factor. This leads to the formation of new blood vessels (neo-angiogenesis) that are often structurally abnormal, fragile, and excessively permeable, contributing to perilesional edema. The disruption of the BBB further enhances vascular leakage and cerebral edema, perpetuating a cycle of damage. While the primary injury in RBN is widely accepted to be vascular damage, leading to secondary damage in the brain parenchyma, the interplay of various molecular factors continues to be investigated (6). The prevailing hypothesis for RBN pathogenesis is primary microvascular damage followed by secondary parenchymal degeneration; however, the precise molecular interactions are still being elucidated.

Research into the function and regulatory mechanisms of long non-coding RNAs (lncRNAs) in cerebrovascular pathologies following central nervous system (CNS) injury suggests a potential role for these molecules in mediating vascular homeostasis under pathophysiological conditions, which might also be relevant in understanding radiation-induced vascular damage (9).

### 3.2 Glial cell dysfunction and neuroinflammation: new findings

The glial cell injury theory proposes that radiation directly injures the brain parenchyma, particularly glial cells such as oligodendrocytes, leading to demyelination and subsequent damage to blood vessels (6). While the vascular injury hypothesis is more widely accepted, the role of glial cell dysfunction in the pathogenesis of RBN is significant. Radiation exposure can activate astrocytes and microglia, the resident immune cells of the brain (10). This activation triggers the release of various pro-inflammatory cytokines, including tumor necrosis factor-α (TNF-α) and interleukin-6 (IL-6), as well as chemokines like C-X-C motif chemokine ligand 12 (CXCL12), which can attract other inflammatory cells expressing its receptor, C-X-C chemokine receptor type 4 (CXCR4) (11). This cascade of events results in neuroinflammation and demyelination, further contributing to the development of RBN (6).

Oxidative stress and the generation of free radicals also play a critical role in accelerating neuronal apoptosis following radiation (12). Microglia, when activated, produce pro-inflammatory cytokines such as TNF-α and IL-6, which contribute to the inflammatory environment (6, 13).These cytokines are known to play important roles in immune responses and can mediate cellular injury processes through the generation of reactive oxygen species(ROS) (6). Research has shown that elevated levels of IL-6 and TNF-α are associated with greater neurological damage in conditions like stroke, further highlighting their potential contribution to RBN (14). In human RBN specimens, microglia have been found to express these pro-inflammatory cytokines, indicating their active involvement in the inflammatory process within the necrotic tissue (11).

### 3.3 Crosstalk between inflammation and angiogenesis: current understanding

**Figure 1:**
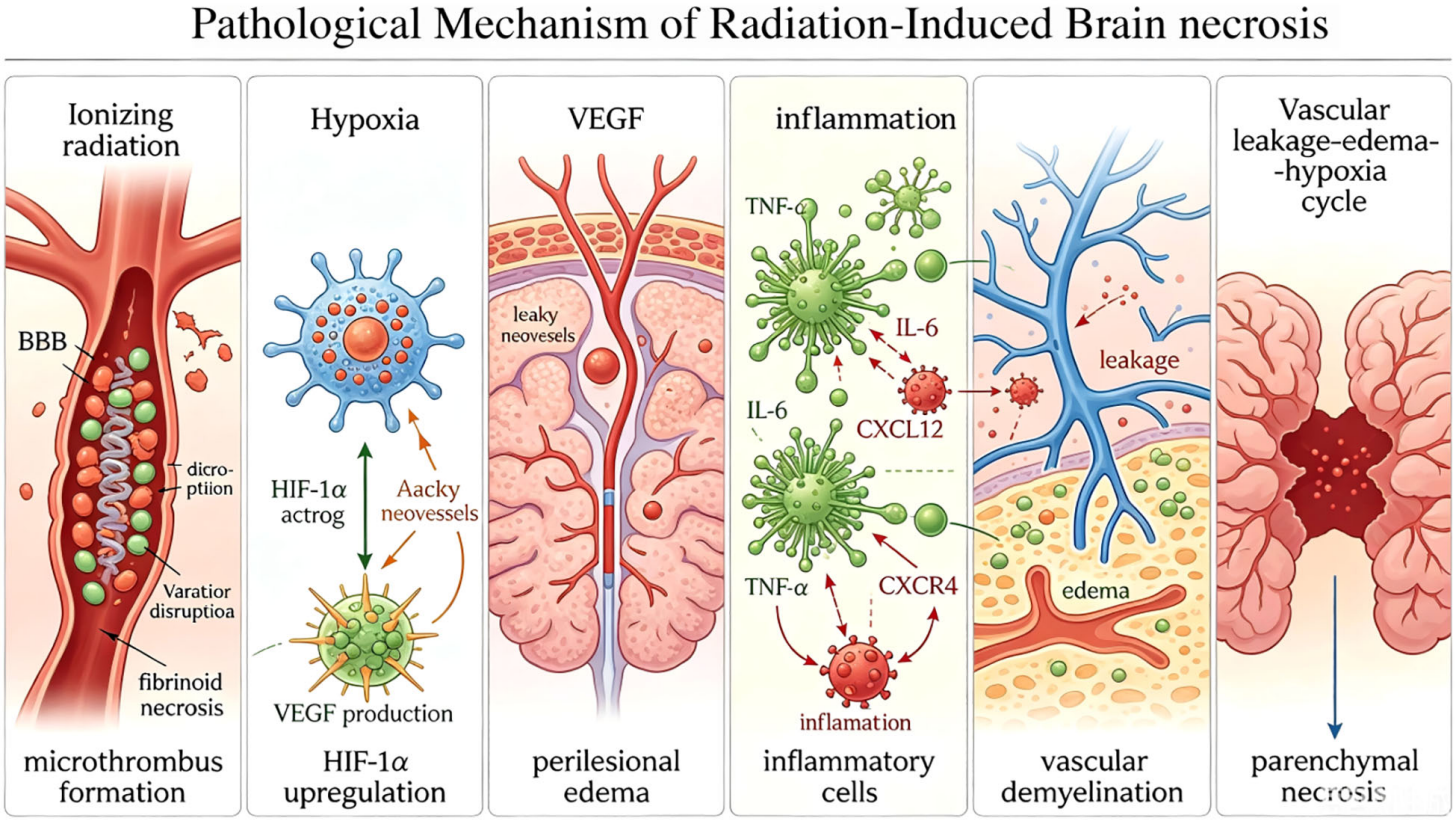
Schematic illustration of the pathogenesis of radiation-induced brain necrosis (RBN). Ionizing radiation directly damages vascular endothelial cells, leading to blood-brain barrier (BBB) disruption, fibrinoid necrosis, and microthrombus formation. Radiation-induced hypoxia upregulates HIF-1α in microglia which in turn stimulates VEGF production in reactive astrocytes VEGF promotes the formation of abnormal, leaky neovessels, exacerbating perilesional edema. Concurrently, radiation activates microglia and astrocytes, triggering the release of pro-inflammatory cytokines (TNF-α, IL-6) and chemokines (CXCL12), which recruit inflammatory cells via CXCR4. This neuroinflammatory environment further amplifies vascular leakage and demyelination. The resulting “vascular leakage–edema–hypoxia” cycle ultimately leads to parenchymal necrosis. BBB, blood-brain barrier; HIF-1α, hypoxia-inducible factor-1α; VEGF, vascular endothelial growth factor; TNF-α, tumor necrosis factor-alpha; IL-6, interleukin-6; CXCL12, C-X-C motif chemokine ligand 12; CXCR4, C-X-C chemokine receptor type 4.

A critical aspect of RBN pathogenesis is the complex interplay between inflammation and angiogenesis (6). The interplay between brain edema, vascular injury, and neuroinflammation creates a self-perpetuating cycle of secondary brain damage (15–17). Brain edema, particularly vasogenic edema resulting from BBB disruption, increases interstitial pressure, impairs microcirculation, and causes mechanical distortion of neuronal tracts (6). This elevated intracranial pressure can lead to tissue hypoxia, compression of vital structures (e.g., brainstem), and ultimately herniation if untreated. Vascular injury exacerbates this process by reducing cerebral blood flow, inducing microthrombus formation, and compromising nutrient delivery to neurons. Neuroinflammation driven by activated microglia and astocytes releasing TNF-α, IL-6, and ROS, directly induces neuronal apoptosis, demyelination, and synaptic dysfunction (12, 15). Clinically, these mechanisms can become fatal in several scenarios: (1) massive cerebral edema leading to transtentorial or tonsillar herniation, particularly in patients with large (>3 cm) necrotic lesions; (2) brainstem necrosis affecting cardiorespiratory centers; (3) intractable seizures progressing to status epilepticus; (4) hemorrhagic transformation of necrotic tissue causing sudden neurological deterioration; and (5) infection (e.g., abscess formation) within necrotic cavities in immunocompromised patients (17, 18).The inflammatory microenvironment created by activated glial cells promotes the secretion of VEGF, a key driver of angiogenesis. However, the newly formed blood vessels are often leaky and dysfunctional, leading to increased vascular permeability, perilesional edema, and hypoxia, thus creating a vicious cycle (6, 19–24). This “vascular leakage-edema-hypoxia” cycle further exacerbates tissue damage. Conversely, vascular damage itself amplifies inflammatory responses by triggering the release of pro-inflammatory mediators. HIF-1α appears to be a central regulator in this crosstalk (11). Hypoxia resulting from vascular damage induces HIF-1α expression in microglia (6, 15).HIF-1α, in turn, upregulates VEGF production in astrocytes, driving the formation of abnormal blood vessels. Furthermore, HIF-1α also regulates the CXCL12/CXCR4 signaling pathway (11, 25).In human RBN specimens, astrocytes express the chemokine CXCL12, while microglia express its receptor CXCR4, suggesting a mechanism by which inflammatory cells are attracted to the perinecrotic area, potentially worsening the edema through the release of pro-inflammatory cytokines (6, 26). Inflammation as well as angiogenesis may participate in the pathophysiology of RBN. This intricate interplay between vascular injury and neuroinflammation, mediated by factors like HIF-1α and the CXCL12/CXCR4 axis, underscores the complexity of RBN pathogenesis (11) (**Figure 1**).

## 4 Progress in prevention strategies for radiocerebral necrosis

### 4.1 Comparative risk assessment: whole brain radiotherapy vs. focal techniques

Before discussing individual advanced modalities, it is important to contextualize the baseline risks associated with conventional radiotherapy approaches. WBRT historically used for multiple brain metastases, delivers low-dose radiation (typically 30 Gy in 10 fractions) to the entire brain. While effective for micrometastatic disease, WBRT is associated with a lower risk of focal RBN (incidence:3-8%) but significant diffuse white matter injury and neurocognitive decline. The multifactorial nature of post-WBRT injury complicates attribution to frank necrosis versus radiation leukoencephalopathy. In contrast, focal radiation delivery techniques – including stereotactic radiosurgery (SRS), fractionated SRS (fSRS), and SRT, which deliver high biologically effective doses to the tumor with rapid dose fall-off outside the target. These techniques achieve superior tumor control but carry a substantially higher risk of focal RBN, ranging from 5–25% depending on lesion size, location, and prior radiation history. The risk-benefit trade-off is clear: focal techniques maximize tumor control at the cost of increased focal necrosis risk, whereas WBRT reduces focal necrosis risk but at the expense of cognitive function. The comparison of WBRT vs. Focal Techniques for RBN Risk was showed in **Table 1**.

**Table 1:** Comparison of whole brain radiotherapy vs. focal techniques for RBN risk.

Parameter	WBRT	Focal techniques (SRS/fSRS/SRT)
RBN incidence	3-8% (typically diffuse, not focal)	5-25% (lesion-specific)
Primary toxicity	Leukoencephalopathy, cognitive decline	Focal necrosis, edema
Typical dose	30 Gy/10 fractions	15–24 Gy single fraction; 24–30 Gy/3–5 fractions
Indication	Multiple (>10-15) metastases, leptomeningeal disease	Oligometastases (1–4 lesions), small-volume disease
Cognitive outcome	Worse (decline at 3–6 months)	Better (preserved)

Significant advancements have been made in strategies aimed at preventing the development of RBN, focusing on refinements in radiotherapy techniques (summarized in **Table 2**) and the exploration of pharmacological interventions (summarized in **Table 3**).

**Table 2:** Comparison of radiotherapy modalities in RBN prevention.

Modality	Mechanism of prevention	Key findings from recent studies	Advantages	Limitations	Future directions
IMRT	Reduces high-dose “hotspots” in sensitive regions	Decreased incidence of RBN and TLN in NPC patients (33)	Greater sparing of normal brain tissue (27)	Risk still exists, especially with skull-base or intracranial invasion (33)	Further optimization of dose constraints for specific brain regions
fSRS	Delivers radiation in multiple fractions, allowing for repair of normal tissue (39)	Lower RBN risk compared to single-fraction SRS, especially for larger lesions	Reduced toxicity for larger volumes	Certain brain regions (e.g., Deep-Periventricular) may still be at higher risk (73)	More prospective randomized trials comparing to single-fraction SRS (45)
FLASH RT	Ultra-high dose rate minimizes oxidative damage to normal tissues	Preserves hippocampal neurons and reduces cognitive deficits in preclinical studies (56, 57); alters ROS dynamics	Significant normal tissue sparing	Still in early stages of clinical translation (56, 57); mechanisms not fully understood	Wider clinical application and further elucidation of mechanisms (74)
Proton Therapy	Bragg peak minimizes exit dose	Reduced volume of normal brain receiving low-to-intermediate doses (64)	Superior sparing of critical structures	Range uncertainty; cost; limited availability	Clinical trials quantifying RBN reduction
TRT	Molecularly targeted systemic delivery	Lower RBN risk than re-irradiation; emerging trial data (67, 68)	Highly targeted; potential for theranostics	Off-target uptake; need for predictive biomarkers	Prospective studies on RBN incidence

**Table 3:** Pharmacological agents in RBN prevention.

Agent	Mechanism of action (Proposed or established)	Key findings from recent studies	Current status (prophylaxis/treatment)	Limitations/challenges
Bevacizumab	Inhibits VEGF, reducing vascular leakage (24, 72)	40% reduction in RBN rates in NSCLC patients with prophylactic use (24)	Shows promise for prophylaxis in high-risk groups; established treatment (24)	Concerns about optimal duration and recurrence after discontinuation (24)
NGF	May protect oligodendrocytes and neurons; promotes functional recovery	More effective than corticosteroids in treating TLN (76); improves cognitive function	Primarily investigated for treatment; potential for prophylaxis (75, 76)	Challenges in delivery and potential immunogenicity
GM1	Neuroprotective effects against radiation-induced brain injury (75); may reverse RBN (33)	Preclinical evidence of radioprotection (75)	Primarily investigated for treatment; potential for prophylaxis	Limited clinical data specifically for RBN prophylaxis
Edaravone	Free radical scavenger; reduces oxidative stress and inflammation (82)	Improved edema and neurological symptoms in RBN patients (82)	Primarily investigated for treatment; potential for prophylaxis (82)	Further research needed to establish prophylactic efficacy and optimal dosing
Boswellia serrata	Anti-inflammatory; inhibits 5-lipoxygenase (77, 79)	Reduced edema and steroid requirement in recent case series (78)	Emerging treatment (corticosteroid-sparing)	Limited prospective data; optimal formulation unclear
Tamoxifen	Protein kinase C inhibitor; reduces glial activation (81)	Preclinical: prevents glial activation and oxidative stress	Preclinical only; not yet clinical	Requires human trials for RBN specifically

### 4.2 Enhancements in radiotherapy modalities

#### 4.2.1 Intensity-modulated radiotherapy for risk reduction

IMRT represents a significant advancement in radiation delivery, allowing for the optimization of radiation doses to irregularly shaped volumes while minimizing exposure to surrounding normal tissues (27–30). In the context of head and neck cancer, IMRT has demonstrated the ability to spare critical structures like salivary glands, the esophagus, optic nerves, the brainstem, and the spinal cord (27, 31, 32). This principle of normal tissue sparing is crucial in preventing RBN following intracranial radiotherapy. By reducing high-dose “hotspots” in sensitive brain regions, such as the temporal lobes, IMRT has contributed to a decline in both the incidence and severity of RBN over the past two decades (33, 34). Specifically, for NPC patients, where the proximity of the nasopharynx to the cerebrum makes the temporal lobes particularly vulnerable, IMRT has shown a reduction in the incidence of temporal lobe necrosis (TLN), a common form of RBN. Studies comparing IMRT to older techniques like two-dimensional conventional radiotherapy (2D-RT) have demonstrated a significant decrease in TLN incidence with IMRT (35, 36). While the exact incidence of TLN can vary due to factors like follow-up duration and imaging advancements, IMRT has undoubtedly improved outcomes by allowing for stricter dose limitations to the temporal lobes (33). Ideally, to minimize the risk of RBN, single-fraction doses should be kept below 12 Gy (33).

#### 4.2.2 Fractionated stereotactic radiosurgery and its preventive role

fSRS involves delivering the total radiation dose in multiple smaller fractions (typically 3–5 fractions) over several days, as opposed to a single high-dose fraction in traditional SRS (37–39). This fractionation strategy has been shown to lower the risk of RBN is by approximately 30% compared to single-fraction SRS (38–40). The radiobiological rationale behind this reduction lies in the radiobiological principle that fractionating the dose allows normal brain tissues more time to repair sublethal damage between fractions, while still delivering a tumoricidal dose to the target (41–43). Consequently, fSRS has become a preferred treatment option for patients with a limited number of brain metastases, especially for larger lesions, demonstrating better neurocognitive outcomes relative to WBRT (44). Although fSRS generally reduces RBN risk (45), a *post-hoc* analysis of fSRS data indicated that lesions located in the Deep-Periventricular region and in close proximity to stem cell niches might still carry a higher risk of RBN (4, 46, 47),underscoring the importance of careful treatment planning. Dose-volume metrics, such as the volume of normal brain receiving at least 12 Gy (V12Gy), remain important predictors of RBN risk even after fSRS (4, 48).Ongoing research, including prospective randomized trials comparing fSRS with single-fraction SRS, aims to further define the optimal fractionation regimens for minimizing RBN risk while maintaining effective tumor control (37, 49).

Regarding the relative efficacy of hyperfractionation (≥2 fractions/day with smaller doses per fraction) versus hypofractionation (3–5 fractions over days to weeks) (50), no definitive consensus has been established (51).Current evidence suggests that hypofractionated SRS (hfSRS), typically delivering 24–30 Gy in 3–5 fractions, is more commonly used in clinical practice and has demonstrated favorable toxicity profiles (52–54). Hyperfractionation (e.g., 1.2 Gy twice daily) has been studied primarily in the context of re-irradiation, but comparative data between these two approaches specifically for edema mitigation are lacking (52–54). A recent meta-analysis found no significant difference in RBN incidence between fractionation schedules delivering biologically effective doses (BED) below 50 Gy (55), suggesting that total BED may be more important than fractionation pattern per dose (56, 57). Further prospective randomized trials are needed to directly compare hyperfractionation versus hypofractionation.

#### 4.2.3 FLASH ultra-high-dose-rate radiotherapy

The underlying mechanisms for this normal tissue sparing effect are still being investigated but likely involve altered ROS dynamics and rapid oxygen depletion within normal cells, making them more resistant to radiation damage (58–60). Furthermore, FLASH RT appears to induce less neuroinflammation in the brain and may even preserve synaptic plasticity (61). Emerging evidence from preclinical studies has begun to elucidate the specific vascular protective effects of FLASH RT. Critically, FLASH irradiation preserves the structural integrity of the neurovascular unit, a key component whose disruption is central to the pathogenesis of RBN and vascular damage (62). Using a mouse model, Dokic and colleagues demonstrated that helium ion FLASH RT significantly reduced double-strand breaks and preserved CD31^+^ microvascular density compared with standard dose-rate irradiation, indicating protection of the neurovascular endothelium (62). This preservation of vascular architecture was associated with reduced activation of CD68^+^ iNOS^+^ phagocytic microglia/macrophages, suggesting that FLASH RT mitigates the radiation-induced inflammatory cascade that typically follows vascular injury (48, 57, 59, 61, 62). Complementary studies have shown that, unlike conventional radiotherapy, FLASH RT does not compromise BBB integrity, as evidenced by maintained expression of tight junction proteins claudin-5 and occludin, preserved aquaporin-4 localization, and unaltered endothelial nitric oxide synthase expression. Mechanistically, the vascular-sparing effect of FLASH RT has been linked to reduced phosphorylation of myosin light chain (MLC), thereby limiting endothelial cell contraction and subsequent immune cell infiltration; notably, pharmacological inhibition of MLC kinase recapitulates the FLASH effect under conventional dose-rate conditions. A comprehensive review by Chow and Ruda synthesizes these findings, noting that the FLASH effect on blood vessels, encompassing preservation of endothelial integrity, reduced apoptosis, and maintenance of tight junction proteins represents a distinct biological process separable from the physicochemical oxygen depletion hypothesis (63). While primarily in preclinical stages, the potential of FLASH RT to significantly improve the therapeutic window in radiotherapy by reducing normal tissue toxicity and preventing complications like RBN—through both direct neuroprotection and preservation of the vascular compartment is substantial (52–54).Clinical translation of FLASH RT is underway, with initial trials showing feasibility and promising results, warranting further investigation into its broad application in cancer treatment.

#### 4.2.4 Emerging modalities: proton therapy and TRT

Beyond photon-based techniques and FLASH, other novel radiotherapy modalities offer theoretical advantages in sparing normal brain tissue and reducing RBN risk. Proton therapy utilizes the physical property of the Bragg peak, depositing the maximum radiation dose at a defined depth with minimal exit dose beyond the target. This unique dosimetric profile allows for superior sparing of critical structures (e.g., hippocampus, brainstem, optic pathways) compared to even the most advanced photon-based IMRT or Volumetric Modulated Arc Therapy (VMAT). In the treatment of primary brain tumors, the transition from photon to proton therapy has been associated with a reduced volume of normal brain receiving low-to-intermediate radiation doses, which is expected to translate into a lower incidence of late toxicities, including RBN (64). However, clinical data specifically quantifying RBN reduction with protons remain limited, and factors such as range uncertainty, patient setup variability, and the potential for increased relative biological effectiveness (RBE) at the distal edge require continued optimization (65). Targeted Radionuclide Therapy (TRT) represents a paradigm shift from external beam to systemic, molecularly targeted radiation delivery (66). TRT uses radiolabeled ligands (e.g., peptides, antibodies, small molecules) that bind to tumor-specific receptors (e.g., somatostatin receptors in meningiomas, integrins or amino acid transporters in gliomas), delivering cytotoxic radiation directly to tumor cells while relatively sparing surrounding normal brain. For primary brain tumors, emerging clinical trials are investigating TRT with agents such as ¹^77^Lu-DOTATATE, ²¹³Bi-DOTATATE, and ^90^Y-labeled compounds (67, 68). The potential for RBN with TRT appears lower than with focal external beam re-irradiation, given its more targeted delivery, but risks include off-target uptake in normal tissues and, rarely, radiation necrosis in regions of high tracer accumulation (e.g., tumor margins with high receptor density). Larger prospective studies are needed to define the true incidence of RBN following TRT and to identify predictive biomarkers. The integration of TRT into neuro-oncology represents a key frontier, enabled by advances in theranostics that pair diagnostic imaging (e.g., ^68^Ga-DOTATATE PET) with therapeutic radionuclides (69–71). Both proton therapy and TRT hold significant promise for reducing iatrogenic brain injury, including RBN, but their widespread adoption is currently limited by high costs, infrastructure requirements (protons), and the need for further efficacy and safety data in large patient cohorts.

### 4.3 Pharmacological prophylaxis

#### 4.3.1 Bevacizumab as a preventive agent: latest evidence

Bevacizumab, a humanized monoclonal antibody that targets vascular endothelial growth factor (VEGF), has shown promise in reducing the incidence of RBN by inhibiting vascular leakage. VEGF plays a critical role in the development of abnormal, leaky blood vessels associated with RBN. By blocking VEGF, bevacizumab can normalize the vasculature and reduce edema (72). A retrospective study in non-small cell lung cancer (NSCLC) patients with brain metastases reported a significant 40% reduction in RBN rates with the prophylactic use of bevacizumab concurrent with SRS (24).

#### 4.3.2 Exploring the prophylactic use of neuroprotective agents (NGF, GM1, edaravone)

Several neuroprotective agents are being investigated for their potential to mitigate radiation-induced neuronal damage and prevent RBN (**Table 3**). Nerve growth factor (NGF) is a neurotrophin that has shown promise in treating established radiation necrosis and improving cognitive function. A phase II clinical trial demonstrated that NGF was more effective than corticosteroids in recovering from TLN with minimal side effects (75, 76). Monosialotetrahexosylganglioside (GM1), a ganglioside found in the CNS, has also shown neuroprotective effects against radiation-induced brain injury in preclinical studies and has been reported to have potency in reversing RBN. Edaravone, a free radical scavenger, has demonstrated therapeutic benefits in patients with radiation-induced brain necrosis by reducing edema and improving neurological symptoms in randomized controlled trials Its antioxidant and anti-inflammatory properties and potential to inhibit ferroptosis, a form of cell death, suggest a possible role in prophylaxis by reducing oxidative stress and neuronal damage. Boswellia serrata exerts anti-inflammatory effects chiefly through 5-lipoxygenase suppression; recent case series demonstrate its capacity to mitigate cerebral edema and lower reliance on concomitant corticosteroids, rendering it a promising corticosteroid-sparing emerging therapeutic candidate, albeit constrained by scarce prospective clinical evidence and undefined optimal preparation specifications (77–80). As a protein kinase C inhibitor capable of restraining glial activation, tamoxifen has displayed preclinical efficacy in suppressing glial overactivation and alleviating oxidative stress after brain damage, yet relevant research remains confined to preclinical investigations without human clinical validation, necessitating dedicated prospective human trials targeting RBN to confirm its clinical utility (81).While the evidence for the prophylactic use of these neuroprotective agents is still evolving, their therapeutic efficacy in managing RBN warrants further investigation into their potential preventive roles.

## 5 Therapeutic advances in managing symptomatic radiation necrosis

When RBN becomes symptomatic, various therapeutic options are available, ranging from established first-line treatments to emerging second-line and precision interventions.

### 5.1 First-line treatment modalities: efficacy and limitations

#### 5.1.1 The role of corticosteroids in contemporary management

Corticosteroids, such as dexamethasone, have long been a mainstay in the initial management of symptomatic radiation necrosis. They provide short-term relief from cerebral edema by reducing inflammation and vascular permeability (83). However, the long-term use of corticosteroids is associated with significant side effects, including hyperglycemia, increased risk of infections, myopathy, and iatrogenic Cushing’s syndrome. While corticosteroids can effectively alleviate symptoms related to edema, their impact on the underlying necrotic process is limited, and symptoms often recur upon discontinuation. Consequently, there is a growing need for corticosteroid-sparing alternatives for the long-term management of RBN. Emerging evidence suggests that bevacizumab might be more efficacious than corticosteroids in treating RBN.

#### 5.1.2 Bevacizumab in treating established radiation necrosis: recent outcomes

Bevacizumab has emerged as a highly effective treatment for symptomatic radiation necrosis. Administered intravenously at doses ranging from 5 to 7.5 mg/kg every 2 to 3 weeks, bevacizumab has demonstrated the ability to reduce the size of necrotic lesions and improve Karnofsky Performance Status scores in approximately 80% of patients. Clinical trials and case series have reported significant radiographic responses, with median volume reductions of 50% or more (19–22, 78, 84–91). Patients often experience improvement in neurological symptoms and a reduction in the need for corticosteroids. Regarding established guidelines for dosing and duration, the 2022 DEGRO guidelines (92) recommend intravenous bevacizumab at 5–7.5 mg/kg every 2–3 weeks for a typical course of 2–6 cycles. A common regimen from prospective trials is 7.5 mg/kg every 3 weeks for 4 cycles. Clinical consensus suggests that treatment should continue until symptomatic improvement and radiographic response (typically edema reduction and decreased contrast enhancement) are achieved. However, there is no universally agreed-upon optimal duration, and decisions are often individualized based on response and tolerability. However, a significant challenge with bevacizumab treatment is the high rate of recurrence, with 50% to 70% of patients experiencing regrowth of the necrotic lesion after discontinuation, likely due to the irreversible nature of the underlying vascular damage. Despite this, bevacizumab remains a crucial first-line treatment option for symptomatic RBN, often leading to substantial short-term benefits.

### 5.2 Second-line and emerging therapeutic options

#### 5.2.1 Hyperbaric oxygen therapy: current evidence base

Hyperbaric oxygen therapy (HBOT) involves breathing pure oxygen in a pressurized environment, which enhances tissue oxygenation and can promote angiogenesis, potentially aiding in the repair of radiation-damaged tissues (93, 94).Studies have shown that HBOT can stabilize or improve symptoms in approximately 60% of patients with radiation necrosis (83, 95). A retrospective review of patients with symptomatic brain RBN treated with HBOT reported clinical improvement in 92% of cases and radiological improvement or stability in most patients (96, 97). While large-scale, double-blind placebo-controlled trials are lacking, case studies and prospective studies suggest a benefit. The most common side effect associated with HBOT is middle ear barotrauma. HBOT is considered a safe and potentially effective second-line treatment option for radiation necrosis, particularly in cases where other therapies have failed or are contraindicated (98).

#### 5.2.2 Laser interstitial thermal therapy: advancements and applications

Laser interstitial thermal therapy (LITT) is a minimally invasive, image-guided technique used to ablate necrotic foci in the brain (99–105).

Studies have demonstrated that LITT can lead to edema resolution and neurological improvement in patients with radiation necrosis, often within 7 weeks (106–113).A meta-analysis comparing bevacizumab and LITT for the treatment of RBN found no significant differences in radiological improvement or steroid wean-off rates, suggesting that LITT is a comparable treatment option. Furthermore, LITT has shown efficacy in managing corticosteroid-refractory RBN, with high rates of symptom improvement and radiological stability (114). This minimally invasive approach offers an alternative to surgical resection, particularly for lesions in deep or eloquent brain areas.

#### 5.2.3 Targeting microglia: the promise of CSF1R inhibitors

Microglia, the resident immune cells of the brain, play a significant role in the inflammatory processes associated with radiation necrosis (6).

Following radiation, microglia become activated and release pro-inflammatory cytokines that contribute to tissue damage (6). Colony-stimulating factor 1 receptor (CSF1R) inhibitors, such as PLX3397, are emerging as potential therapeutic agents for RBN by targeting and depleting these activated microglia (115, 116). In animal models of various neurological conditions, CSF1R inhibitors have shown promise in reducing inflammatory cytokine release and improving cognitive function (117, 118).

While research on the direct application of CSF1R inhibitors in treating radiation necrosis is still in its early stages, the preclinical findings suggest that targeting microglia and the associated neuroinflammation could be a novel and effective therapeutic strategy for this challenging condition (119).

#### 5.2.4 Surgical resection

Surgical resection (necrotomy) remains a significant option as second-line or third-line treatment, particularly for refractory cases where medical therapies (corticosteroids, bevacizumab, HBOT) have failed or are contraindicated (8, 120). The clinical utility of necrotomy includes (1): definitive tissue diagnosis to exclude tumor recurrence when imaging is equivocal (2); immediate relief of mass effect from large necrotic lesions causing life-threatening herniation (3); reduction of corticosteroid dependence in patients with steroid-refractory or steroid-intolerant RBN; and (4) treatment of cystic or hemorrhagic necrotic lesions that are poorly responsive to pharmacotherapy. Case series report that surgical resection leads to symptomatic improvement in 60–80% of patients with refractory RBN, with acceptable perioperative risks when performed in experienced centers (120, 121).However, the pathological challenges outlined above, namely obliterative fibrosis, inherently fragile vasculature, and defective hemostatic capacity, demand precise, high-caliber microsurgical technique alongside continuous intraoperative neuromonitoring throughout the procedure. For deep or eloquent lesions, minimally invasive alternatives such as LITT may be preferred.

## 6 Innovations in imaging for early detection and differential diagnosis

Accurate diagnosis and differentiation of radiation necrosis from tumor recurrence are crucial for guiding appropriate management strategies. Advancements in imaging techniques, particularly functional and metabolic MRI, have significantly improved diagnostic accuracy.

### 6.1 Functional MRI techniques: enhancing diagnostic accuracy

#### 6.1.1 Magnetic resonance spectroscopy in early identification

Magnetic Resonance Spectroscopy (MRS) is a non-invasive technique that provides information about the metabolic composition of brain tissue (122–126). In the context of RBN, MRS can detect characteristic metabolic changes that differ from those seen in tumor recurrence. Reduced ratios of N-acetyl aspartate (NAA) to creatine (Cr) typically indicate neuronal injury, while elevated ratios of choline (Cho) to Cr reflect increased cellular turnover and gliosis, which are features of RBN (6, 126). These metabolic markers can often be detected earlier than the structural changes visible on conventional MRI, enabling earlier diagnosis (6). However, differentiating tumor recurrence from RBN using MRS alone is often insufficient for several reasons. First, the two conditions frequently coexist, particularly in the margins of irradiated tumor beds, leading to mixed metabolic profiles. Second, interpretation is highly dependent on timing of examination (early post-radiation changes versus late RBN) and the selected region of interest (ROI), as sampling errors can lead to false conclusions (6, 127).Third, tumoral necrosis and sterile RBN can produce overlapping metabolite patterns. Consequently, MRS used in isolation has limited diagnostic accuracy (sensitivity ~70%, specificity ~65%).Therefore, rather than relying on MRS alone, combining it with PWI and PET, as discussed in the following sections, leads to a more definitive and reliable diagnosis (128). A multimodality approach integrating MRS, PWI, and amino acid PET has been shown to improve diagnostic accuracy to >90% in recent meta-analyses (128).When there is uncertainty in differentiating RBN from tumor progression based on standard MRI, MRS, along with other advanced imaging modalities, can significantly improve diagnostic confidence.

#### 6.1.2 Perfusion-weighted imaging for differentiation

Perfusion-Weighted Imaging (PWI) is an MRI technique that assesses cerebral blood volume and flow (6, 129–131). It plays a vital role in distinguishing radiation necrosis from tumor recurrence. In radiation necrosis, the relative cerebral blood volume (rCBV) is typically lower compared to tumor recurrence, which often exhibits increased vascularity and hyperperfusion (6, 132). Studies have shown that lower rCBV in a lesion has a high diagnostic specificity (around 85%) for radiation necrosis compared to tumor recurrence (126, 133, 134). Similar to MRS, PWI is considered an important ancillary technique that can enhance the accuracy of differentiating these two conditions, especially when conventional MRI findings are equivocal (135).

### 6.2 The role of metabolic imaging: PET/CT with advanced tracers

Positron Emission Tomography (PET/CT) is a metabolic imaging technique that uses radiotracers to assess cellular activity (136–139). In the diagnosis of RBN, 18F-fluorodeoxyglucose (18F-FDG) PET/CT can show hypometabolism in the area of necrosis, as necrotic tissue has reduced glucose uptake (6, 138). However, 18F-FDG PET/CT has limitations due to high glucose metabolism in the normal brain and the potential for FDG uptake in inflammatory processes, which can occur in both RBN and tumor recurrence (6, 139, 140). To overcome these limitations, PET imaging with amino acid tracers, such as 11C-methionine (11C-MET) and 18F-fluoroethyltyrosine (18F-FET), has proven more effective (141). These tracers show high uptake in areas of tumor recurrence due to increased protein synthesis, whereas their uptake is typically low in RBN, which is characterized by tissue breakdown and reduced metabolic activity (6). The sensitivity and specificity of amino acid PET in differentiating RBN from tumor recurrence can be as high as 85% and 88%, respectively (141–143).

## 7 Updated international guidelines for the management of radiation necrosis

International guidelines for the management of radiation necrosis are continuously updated to reflect the latest research and clinical experience. The 2022 guidelines from the German Society for Radiation Oncology (DEGRO) provide a framework for the diagnosis and treatment of this condition, emphasizing a multidisciplinary and personalized approach (92).

### 7.1 The 2022 DEGRO guidelines and beyond: a multidisciplinary approach

The 2022 DEGRO guidelines advocate for a multistep diagnostic workflow that integrates clinical history, advanced imaging techniques (including MRI and PET), and the consideration of biopsy when necessary to definitively differentiate radiation necrosis from tumor recurrence (92). This multidisciplinary approach involves collaboration between radiation oncologists, neuro-oncologists, neuroradiologists, and neurosurgeons to ensure accurate diagnosis and optimal management (92). The guidelines recognize the challenges in distinguishing RBN from tumor progression based solely on conventional imaging and highlight the importance of utilizing functional and metabolic imaging techniques to improve diagnostic accuracy (92).

### 7.2 Towards personalized diagnostic and treatment strategies

The treatment recommendations outlined in the DEGRO guidelines emphasize a personalized approach based on the individual patient’s clinical presentation and the severity of symptoms (92). For asymptomatic cases of suspected radiation necrosis, close clinical and radiological monitoring is recommended without aggressive intervention. For symptomatic cases, bevacizumab (19–24, 84–92, 144–158). or laser interstitial thermal therapy (LITT) are considered first-line treatment options, demonstrating efficacy in reducing edema and improving neurological symptoms. Surgical resection (8, 92, 159–161) is generally reserved for refractory cases where medical therapies have failed to provide adequate relief or when there is diagnostic uncertainty and a tissue diagnosis is required. The guidelines underscore the importance of accurate diagnosis to tailor the treatment approach effectively, recognizing that the optimal management strategy may vary depending on factors such as the location and size of the lesion, the patient’s overall clinical condition, and the presence of comorbidities (92).

## 8 Navigating challenges and charting future directions in radiocerebral necrosis

Despite the advancements in understanding and managing radiation necrosis, several challenges remain, and ongoing research continues to explore new avenues for prevention and treatment.

### 8.1 Key areas for mechanistic research: unanswered questions

Further research is needed to clarify the complex spatiotemporal interactions between inflammation and vascular injury in the pathogenesis of radiation necrosis (6). Understanding how these two core mechanisms interact and influence each other over time is crucial for developing more targeted and effective interventions. Exploring novel therapeutic targets, such as the CXCL12/CXCR4 signaling axis and HIF-1α inhibitors, holds significant promise. Preclinical studies have shown that inhibiting HIF-1α with agents like topotecan and blocking the CXCL12/CXCR4 pathway with antagonists like AMD3100 can mitigate the progression of RBN in animal models by reducing inflammation (162). The CXCL12/CXCR4 axis is involved in various cellular processes relevant to RBN, including cell migration, proliferation, angiogenesis, and inflammation (163, 164). Further investigation into the precise roles of these pathways in the context of irradiated brain tissue is warranted to pave the way for future targeted therapies.

### 8.2 Strategies for therapeutic optimization: combination and targeted approaches

Future therapeutic strategies for radiation necrosis may involve the development of synergistic regimens that combine different treatment modalities to target multiple aspects of the disease pathology (165). For example, combining bevacizumab, which primarily targets vascular abnormalities, with agents that specifically inhibit pro-inflammatory cytokines like IL-1β could potentially enhance treatment efficacy (165). The use of nanoparticle-based drug delivery systems could also be explored to achieve more targeted delivery of therapeutic agents to the necrotic tissue, minimizing off-target effects (75). Furthermore, continued investigation into the potential of existing drugs like edaravone (82), possibly in combination with other therapies, is warranted given its antioxidant and anti-inflammatory properties (166). Addressing the challenges associated with growth factor-based therapies, such as NGF, through innovative delivery methods could also unlock their therapeutic potential in RBN (165).

### 8.3 Expanding the horizon of prevention: novel modalities and technologies

Expanding the clinical application of FLASH ultra-high-dose-rate radiotherapy offers a significant opportunity to further minimize the risk of radiation necrosis due to its remarkable normal tissue sparing effects. Additionally, the integration of artificial intelligence (AI) into dosimetric planning could lead to more precise and optimized radiation delivery, further reducing the likelihood of RBN by carefully sculpting the radiation dose to target the tumor while avoiding sensitive brain regions. Exploring the preventive potential of other pharmacological agents, such as tamoxifen, curcumin, and quercetin, which have shown promise in preventing glial cell activation, proliferation, and oxidative stress caused by irradiation, might also be a direction for future research.

## 9 Conclusion

The effective management of radiation necrosis necessitates a collaborative effort across various disciplines, including radiophysics, molecular biology, and clinical medicine. Significant advances in our understanding of the pathogenesis, coupled with innovations in targeted therapies, sophisticated imaging technologies, and refined radiotherapy systems, hold considerable promise for improving outcomes for patients at risk of or affected by this challenging complication. The ongoing evolution of these fields offers the potential to shift the paradigm from primarily focusing on delayed symptom control to achieving early and potentially curative interventions, ultimately enhancing patient survival and significantly improving their quality of life.

Future research should also focus on identifying patient subsets most likely to benefit from proton therapy or TRT in terms of RBN risk reduction, as well as developing standardized imaging criteria for RBN diagnosis following these novel modalities.
